# Binary Solutions of Hyaluronan and Lactose-Modified Chitosan: The Influence of Experimental Variables in Assembling Complex Coacervates

**DOI:** 10.3390/polym12040897

**Published:** 2020-04-13

**Authors:** Federica Vecchies, Pasquale Sacco, Eleonora Marsich, Giuseppe Cinelli, Francesco Lopez, Ivan Donati

**Affiliations:** 1 Department of Life Science, University of Trieste, Via Licio Giorgieri, 5, 34127 Trieste, Italy; fvecchies@units.it (F.V.); idonati@units.it (I.D.); 2 Department of Medicine, Surgery, and Health Sciences, University of Trieste, Piazza dell’Ospitale 1, I-34129 Trieste, Italy; emarsich@units.it; 3 Department of Agricultural, Environmental and Food Sciences (DiAAA), Università degli Studi del Molise, Via De Sanctis, I-86100 Campobasso, Italy; giuseppe.cinelli@gmail.com (G.C.); lopez@unimol.it (F.L.)

**Keywords:** hyaluronic acid, lactose-modified chitosan, complex coacervation, experimental variables, characterization

## Abstract

A miscibility study between oppositely charged polyelectrolytes, namely hyaluronic acid and a lactose-modified chitosan, is here reported. Experimental variables such as polymers’ weight ratios, pH values, ionic strengths and hyaluronic acid molecular weights were considered. Transmittance analyses demonstrated the mutual solubility of the two biopolymers at a neutral pH. The onset of the liquid-liquid phase separation due to electrostatic interactions between the two polymers was detected at pH 4.5, and it was found to be affected by the overall ionic strength, the modality of mixing and the polymers’ weight ratio. Thorough Dynamic Light Scattering (DLS) measurements were performed to check the quality of the formed coacervates by investigating their dimensions, homogeneity and surface charge. The whole DLS results highlighted the influence of the hyaluronic acid molecular weight in affecting coacervates’ dispersity and size.

## 1. Introduction

The perfect balance between attractive and repulsive interactions among oppositely charged polyelectrolytes often results in the formation of a variety of complex structures possessing different physical-chemical and biological properties that potentially could be translated toward clinical applications [1,2].

Concerning drug delivery systems, complex coacervation between oppositely charged polymers is an attractive method to encapsulate and subsequently vehiculate payload therapeutics [3]. The phase behavior of two oppositely charged polymers in a mixture depends primarily on their molecular properties (i.e., molecular weight and charge density), polymers’ weight ratio and physical-chemical features of the surrounding environment (i.e., pH, ionic strength and temperature) [4,5]. A plethora of micro-/nano-structures assembled via complex coacervation are documented in the literature, for instance stimuli responsive nanocapsules made of alginate and chitosan [6,7] or those based on the interaction between chitosan and hyaluronic acid [8,9], polysaccharides and proteins [5,10,11,12], or simple coacervation involving a single polymer and a multivalent ion [13]. 

Hyaluronic acid (HA) is a linear polyanion present in the extracellular matrix of different biological tissues (e.g., eye, skin, articular cartilage) and endowed with important biological features [14,15,16]. CTL is a lactose-derivative of chitosan showing an improved solubility at neutral pH [17] and an interesting bioactivity toward mammalian cells [18,19]. This engineered polysaccharide behaves as a polycation in acidic conditions [17], as a result of which it is prone to promote electrostatic interactions with oppositely charged ions or macromolecules depending on the ionic strength [13,20,21,22].

Studies on the preparation and characterization of binary (i.e., alginate and CTL) and ternary (i.e., alginate, CTL and HA) mixtures composed of natural polyelectrolytes have been conducted in the past [21,23,24], underlying the importance of these polymers in various sectors, in addition to Tissue Engineering and Drug Delivery [25,26,27,28].

Here, the behavior of binary solutions composed of HA and CTL is analyzed in aqueous solutions. Specifically, this work reports for the first time an in-depth investigation about the effects of experimental variables such as ionic strength, pH and hyaluronic acid molecular weight on the complex coacervation process between the two polysaccharides. Besides, with the aim of considering the resulting coacervates as suitable delivery systems, we investigate colloids features, e.g., the surface charge of newly-formed coacervates. In fact, the presence of a net positive or negative charge on colloids’ surfaces ensures formulation stability, a characteristic that is gradually lost when the surface charge gets close to neutrality due to coalescence phenomena. The evaluation of the ζ-potential parameter gives important information on this matter. Overall, since experimental variables are expected to influence resulting colloids features with a potential impact on their final application, this contribution expands current knowledge on the CTL-HA system and provides key guidelines for assembling nano-networks to be used as carrier candidates in the Drug Delivery sector.

## 2. Materials and Methods

### 2.1. Materials

Sodium hyaluronates, HAs, at different molecular weights (see Table 1), were kindly provided by Sigea s.r.l. (Trieste, Italy). CTL (lactose-modified chitosan, CAS Registry Number 2173421-37-7) was kindly provided by biopoLife s.r.l. (Trieste, Italy). CTL was synthetized starting from Chitoceuticals at Heppe Medical Chitosan GmbH, Halle, Germany. The viscosity average molecular weight, ${\bar{M}}_{v}$, of the template chitosan was 380,000. The chemical composition of CTL was determined by ^1^H-NMR, and the results were the following: fraction of acetylated units, F_A_, 0.07, fraction of deacetylated units, F_D_, 0.33 and fraction of lactose-modified units, F_L_, 0.60. According to the determined composition, the corresponding (derived) ${\bar{M}}_{v}$ value of CTL was 910,000. Hepes buffer, acetic acid (AcOH), sodium acetate (AcNa) and sodium hydroxide were purchased from Sigma Aldrich Chemical Co. (Milwaukee, WI, USA). Hydrochloric acid was purchased from Carlo Erba (Milano, Italy). Deionized Milli-Q water was used throughout the experiments.

### 2.2. Determination of Intrinsic Viscosity for HAs and CTL

The intrinsic viscosity [η] of polymers used in this work was determined by means of a CT 1150 Schott Geräte automatic measuring apparatus and a Schott capillary viscometer. In the case of CTL, the same protocol described in [13] was used, and the intrinsic viscosity resulted in 470 mL/g. In the case of HAs, the samples were solubilized using NaCl 0.15 M as a buffer solution. Subsequently, the solutions were filtered through 0.45 μm Millipore (Germany) nitrocellulose filters before the measurement. The intrinsic viscosities were calculated at *T* = 25 °C by analyzing the polymer concentration dependence of the reduced specific viscosity ηspc and of the reduced logarithm of the relative viscosity ln(ηr)c, calculated from Huggins (1) and Kraemer (2) Equations, respectively:

(1) $$\frac{\eta_{sp}}{c}=\left[\eta \right]+k_{1}{\left[\eta \right]}^{2}c$$

(2) $$\frac{ln\;\eta_{r}}{c}=\left[\eta \right]-k_{2}{\left[\eta \right]}^{2}c$$

where *k_1_* and *k_2_* are the Huggins and Kraemer constants, respectively. The resulting intrinsic viscosity values were averaged, and the results are reported in Table 1. In the case of the HA samples, the corresponding viscosity average molecular weight ($\bar{M_{v}}$) was calculated using the Mark-Houwink-Sakurada (MHS) equation (Equation (3)):

(3) $$\left[\eta \right]=K\times \bar{M_{v}^{a}}$$

where the *K* and *a* parameters correspond to 2.63 × 10^−4^ dL/g and 0.81, respectively [29]. 

### 2.3. Miscibility Studies of HA and CTL

The HA samples reported in Table 1 and CTL were dissolved separately in deionized filtered water at a final concentration of 1.5 g/L. The polymers solutions were both prepared at two different pH values, namely 7.4 and 4.5. The polymers were then filtered through 0.45 μm filters. The two polymers were used for the preparation of binary polymer mixtures by mixing different amounts of HA and CTL solutions, hence resulting in different HA weight fractions (*r*HA 0.15 – 0.25 – 0.50 – 0.75). A total polymer concentration of 1.5 g/L was kept constant. The binary solutions were gently stirred for 20 min at room temperature before performing further analyses. The modality of mixing (i.e., CTL solution dropped into HAs solutions during stirring and vice versa), the use of HAs at different molecular weights and the presence of supporting salt (i.e., NaCl 150 - 100 - 75 - 50 - 25 mM) were considered in the investigation of complex coacervation. Hepes and AcOH/AcNa buffers were used to stabilize the pH of the solutions in the case of a final pH of 7.4 and 4.5, respectively. A supporting salt (NaCl)/buffer ratio equal to 15 was kept constant throughout the measurements [21].

### 2.4. Turbidity Measurements

The Transmittance (*T*) of the binary mixtures, both at pH 7.4 and 4.5, was measured at λ = 550 nm with an Ultrospec 2100 pro spectrophotometer (Biochrom). As a blank, the transmittance of separate polysaccharides and that of deionized water was measured as well. At least three replicates were recorded for each sample preparation, and the results were averaged.

### 2.5. Dynamic Light Scattering Analyses

Dynamic Light Scattering (DLS) measurements were performed using a Zetasizer Nano ZS system (Malvern Instruments, UK). The average hydrodynamic diameter and the polydispersity index (PDI) were considered as analytical parameters to shed light on the physical properties of coacervates. Experimentally, the latter were diluted 1:10 v/v in filtered deionized water (pH 4.5), and DLS measurements were performed at *T* = 25 °C, analyzing each sample in triplicate.

DLS was also used to determine the surface charge of samples. Briefly, HAs and CTL were solubilized separately at a final concentration of 1.5 g/L, pH 4.5, as described above. HAs were added to the CTL solution drop-wise, during stirring, in order to obtain solutions with different *r*HA values. The samples were diluted 1:10 in deionized filtered water before ζ-potential analyses. The ζ-potential of the single polymer solutions at pH 4.5 were also recorded. All values are reported as the mean of three replicates.

## 3. Results and Discussion

Several experimental variables have been taken into account in order to study the complex coacervation between HAs and CTL polyelectrolytes. At first, we analyzed the miscibility of CTL and HA90 at pH 7.4 and no ionic strength. In more detail, we investigated the role played by polymer mixing (CTL dropped into an HA90 solution during stirring - indicated as CTL/HA90 - and vice versa, i.e., HA90/CTL) and the final polymers’ weight fraction, indicated as *r*HA for convenience. 

**Figure 1:**
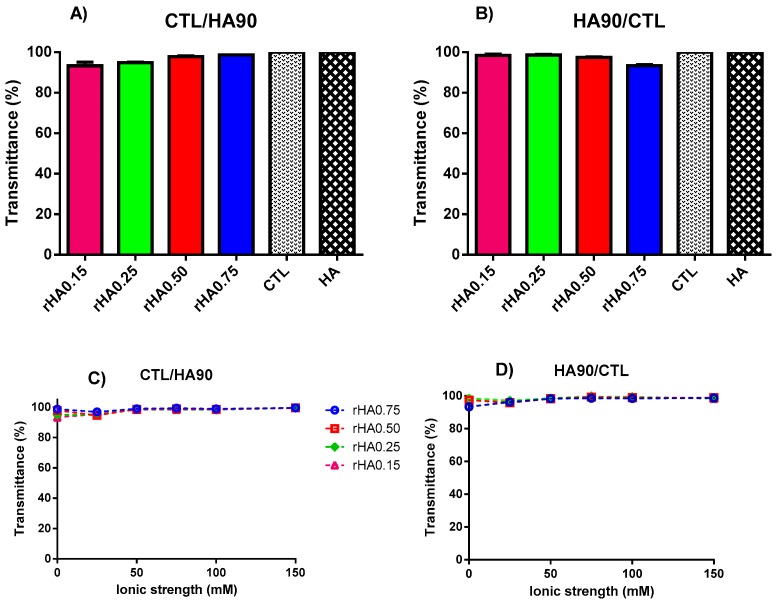
Influence of polymers’ weight fraction and ionic strength on the transmittance for binary solutions of HA90 and CTL prepared at pH 7.4. (**A**,**C**) Formulations prepared by dropping CTL into the HA90 solution. (**B**,**D**) Formulations prepared by dropping HA90 into the CTL solution. The results are reported as the mean (±SD, *n* = 3). CTL and HA are the single polymer solutions used as the controls.

The onset of the turbidity of the system marks the phase transition from disentangled polymers to an associative phase separation, allowing a first indication of coacervates formation [1]. As shown in Figure 1, the transmittance of binary solutions prepared at pH 7.4 is comparable with that recorded for the single polymer solutions and pure distilled water used as the control (*T* ~ 100%). This can be explained by the fact that the pK_a_ of primary and secondary amines of CTL are 6.69 and 5.87, respectively [17]; hence, CTL is mostly uncharged at pH 7.4. This means that inter-chain electrostatic interactions between the two polysaccharides are strongly impaired. Therefore, in these pH conditions, no complex coacervation occurred. 

Next, we increased the complexity of the system by preparing binary mixtures at pH 7.4 in the presence of different amounts of NaCl in order to vary the total ionic strength (*I*). Again, no turbidity was detected while varying polymers’ weight ratios, as the *T* values flattened around 100% (Figure 1C,D). A similar behavior was observed by Donati et al. [24] for binary mixtures of the polyanion alginate and CTL, where the mutual solubility was similarly achieved at a neutral pH and 150 mM of ionic strength.

**Figure 2:**
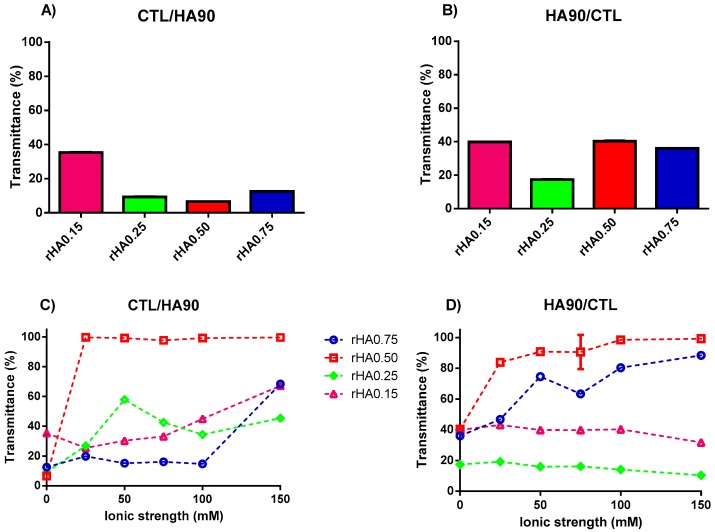
The influence of polymers’ weight fraction and ionic strength on the transmittance for binary solutions of HA90 and CTL prepared at pH 4.5. (**A**,**C**) Formulations prepared by dropping CTL into the HA90 solution. (**B**,**D**) Formulations prepared by dropping HA90 into the CTL solution. The results are reported as the mean (±SD, *n* = 3).

By decreasing the pH to 4.5 and avoiding any addition of supporting salt, a liquid-liquid phase separation occurred, as confirmed by the immediate onset of turbidity (i.e., decrease in transmittance) upon polymer mixing (Figure 2A,B). Under these experimental conditions, the phase separation, with the consequent formation of coacervates, is entropically driven by the release of counterions emerging from the onset of electrostatic interactions between CTL and HA90, the net charge of both polyelectrolytes being -45 ± 1 and 60 ± 4 mV for HA90 and CTL, respectively.

Figure 2C,D reports the analysis of the transmittance as a function of the ionic strength for binary solutions prepared at pH 4.5 and different weight fractions of polymers. At variance with the measurements performed at pH 7.4, ionic strength affects the final liquid-liquid phase separation in this case, albeit to a different extent. In more detail, the formulations prepared without supporting salt manifested a straightforward turbidity with the concomitant presence of few micro-size precipitates. Curiously, the formulations showing *r*HA 0.15 and 0.25 prepared by dropping HA90 into CTL did not display any precipitate when considering the whole ionic strength range from 25 to 150 mM. Our findings are consistent with some previous works [30,31], wherein the presence of 150 mM NaCl allowed for the onset of coacervation after one week, although in our case the phase separation occurred immediately after mixing. However, in other cases it was found that the use of a high salt content prevented simple or complex coacervation [13,32,33,34,35], suggesting that an appropriate balance of repulsive and attractive forces is of paramount importance for coacervates formation.

**Figure 3:**
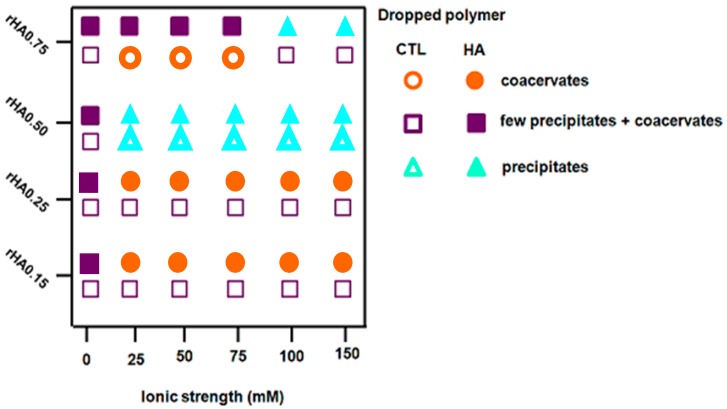
Qualitative representation of the behavior detected for binary solutions after polymer mixing. The pictures are reported as polymers’ weight fraction (*r*HA) as a function of the ionic strength.

Strikingly, we found that the polymer mixing influenced the behavior of final mixtures, since when CTL was dropped into the HA90 solution at the same *r*HA, the presence of precipitates was clearly evident. By changing the content of HA (*r*HA 0.75) for formulations prepared by dropping CTL into HA90, a net turbidity of the system (i.e., low transmittance values) was observed up to *I* = 75 mM without the presence of precipitates. As the ionic strength was further increased, a pronounced increase of the transmittance of the formulations marked, at variance, no straightforward phase separation and the formation of some precipitates. In the case of *r*HA 0.50, the *T* values were found to be very similar to that of pure water for all the ionic strengths analyzed, meaning that no complex coacervation occurred. However, large precipitates were present on the bottom of the vial, ascribed to the lack of true repulsive forces among singular coacervates, causing their coalescence. A qualitative representation of the physical behavior of the different analyzed formulations is reported in Figure 3.

**Figure 4:**
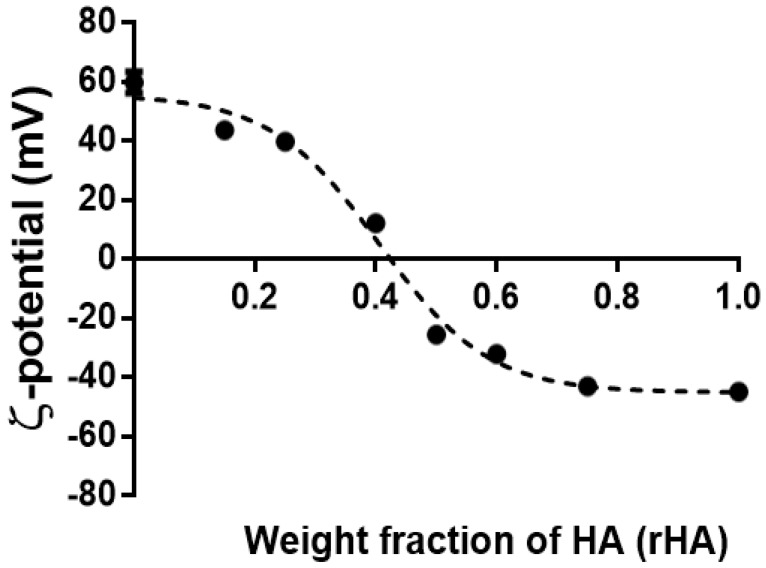
The ζ-potential dependence as a function of the hyaluronic acid weight fraction (*r*HA) of coacervates prepared at pH 4.5, dropping of HA into CTL and negligible ionic strength. The results are reported as the mean (±SD, *n* = 3).

Next, we evaluated the dependence of coacervates’ surface charge on *r*HA by the DLS technique (Figure 4). As expected, the decrement of the positive surface charge was recorded upon increasing the HA amount. Interestingly, our findings highlight the possibility of selecting positive- or negative-surface charged colloids, depending on their final application. The sigmoidal plot profile of the ζ-potential on *r*HA allows for the extrapolation of *r*HA, for which the charge ratio is equal to zero, yielding ~0.43, very close to the 1:1 polymer weight ratio at which coalescence occurred (see above). 

**Figure 5:**
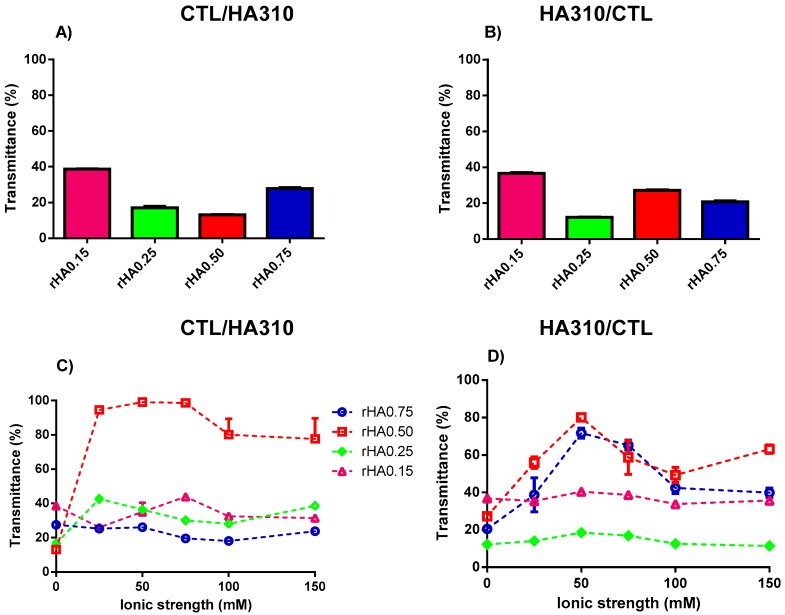
The influence of polymers’ weight fraction and ionic strength on the transmittance for binary solutions of HA310 and CTL prepared at pH 4.5. (**A**,**C**) Formulations prepared by dropping CTL into the HA310 solution. (**B**,**D**) Formulations prepared by dropping HA310 into the CTL solution. The results are reported as the mean (±SD, *n* = 3).

**Figure 6:**
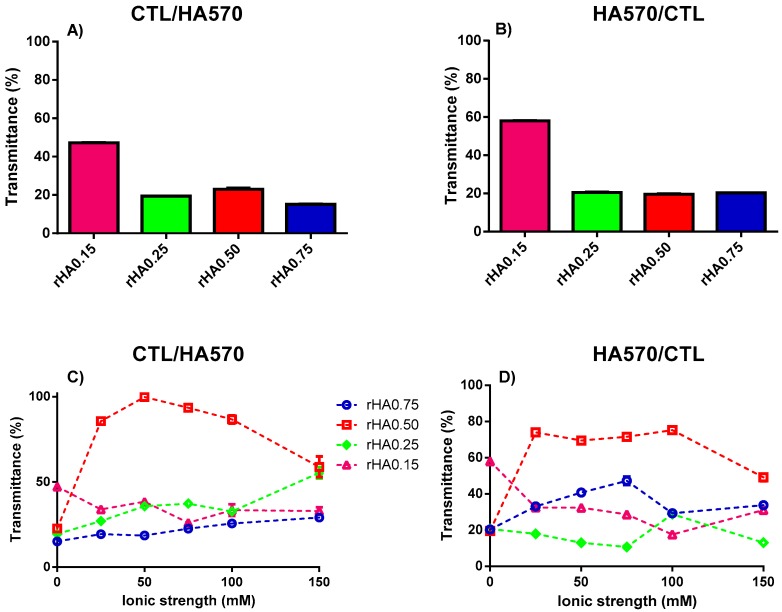
The influence of polymers’ weight fraction and ionic strength on the transmittance for binary solutions of HA570 and CTL prepared at pH 4.5. (**A**,**C**) Formulations prepared by dropping CTL into the HA570 solution. (**B**,**D**) Formulations prepared by dropping HA570 into the CTL solution. The results are reported as the mean (±SD, *n* = 3).

The impact of the HA molecular weight on complex coacervation was also investigated. An increment of turbidity (i.e., low transmittance) was observed for whatever high molecular weight HA (HA310 or HA570) used for the preparation of binary solutions (Figure 5 and Figure 6). The modality of mixing had a negligible effect on *T*, since transmittance remained below 40% for all the formulations investigated. The presence of small amounts of precipitates was also found for binary mixtures using HA310 and HA570. In particular, all CTL-dropped solutions, together with HA-dropped ones showing *r*HA 0.50 and 0.75, displayed the co-existence of both precipitates and coacervates, in line with what was previously reported for HA90. Hence, one can conclude that only in the case of a lower *r*HA (0.15 and 0.25) and the dropping of HA into CTL the formation of insoluble aggregates may be prevented. 

**Figure 7:**
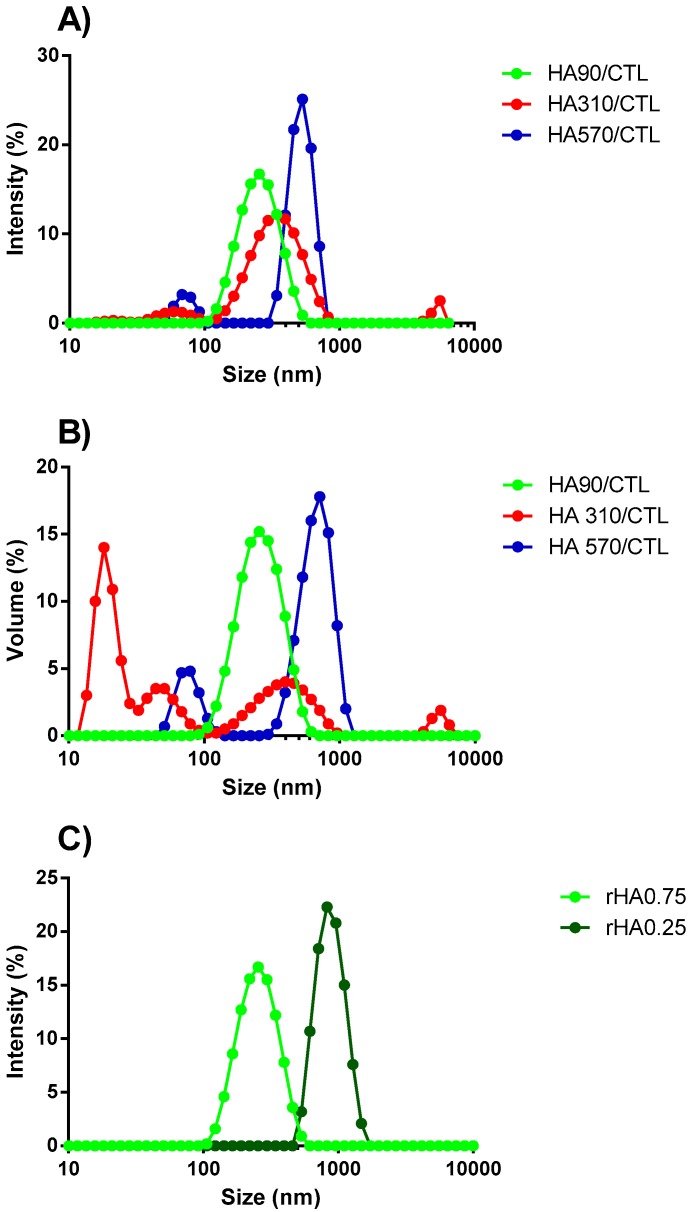
The influence of the HA molecular weight on the size distribution curve profiles of coacervates prepared at pH 4.5, *r*HA 0.75 and the dropping of HAs into CTL. (**A**) The Dynamic Light Scattering intensity and (**B**) volume curve profiles recorded. The HA90/CTL spectrum is reported in green, HA310/CTL in red and HA570/CTL in blue. (**C**) The influence of polymers’ weight fraction (*r*HA) on the physical properties of the resulting coacervates. Coacervates were prepared by dropping HA90 into CTL, pH 4.5 and negligible ionic strength. The light green stands for *r*HA 0.75 and the dark green one for *r*HA 0.25.

Dynamic Light Scattering was used to investigate the influence of the HA molecular weight and *r*HA on the size and polydispersity of the resulting coacervates in the absence of ionic strength and pH 4.5. A comparison of the intensity size and volume distribution curve profiles of three formulations prepared at the same *r*HA but different HA molecular weights is reported in Figure 7. Coacervates prepared with HA90 and *r*HA of 0.75 displayed narrow and symmetric distribution curves, indicating the good homogeneity (PDI = 0.28 ± 0.02) of the resulting colloids, in line with what was reported in the literature for biopolymer-based nanoparticles [8,36], whereas more heterogeneous distribution curves were noticed for systems prepared using HA310 (PDI 0.56 ± 0.05) and HA570 (PDI 0.55 ± 0.21). Two peaks with weak intensities were detected for formulations using HA310, ascribed to the presence of very small scatters (peak around 70 nm) or aggregates (> 1000 nm). More variable results were obtained using HA570. Our findings suggest that by increasing the HA molecular weight, the whole dispersity of coacervates tends to increase, thus diverging from a homogeneous ensemble of colloids. It can be hypothesized that CTL is more prone to accommodate HA90 due to very different molecular weights, i.e., 910,000 vs. 90,000, thus favoring associative phenomena. Hence, HA90 may be envisaged as a cross-linker for CTL [20]. A further consideration stems from the dimensional shift of the main intensity peak toward larger values by increasing the HA molecular weight (Figure 7A), confirming again the role played by the macromolecule dimension in modulating the final size distribution of coacervates. In this view, higher molecular weight HAs lead to steric hindrance, thereby enabling the formation of heterogeneous and larger colloids rather than homogeneous coacervates. These findings are in line with what was reported in the literature for colloidal chitosan/tripolyphosphate (TPP)-based coacervates [37,38], where lower molecular weight chitosans were found to be the best choice for synthetizing mono-dispersed particles [39,40]. The comparison of the intensity and the volume size distributions highlighted that the curves match almost perfectly in the case of coacervates with HA90, whereas the presence of multiple peaks are evidenced when HA310 and HA570 were used (Figure 7B). This evidence confirms the negative role played by higher molecular weight HAs for the final homogeneity of the system. 

DLS analyses were also undertaken to study the influence of *r*HA on the size distribution of coacervates prepared with HA90 (Figure 7C). The hydrodynamic diameter of coacervates changed from around 200 nm (*r*HA 0.75) to almost 1 μm (*r*HA 0.25) by increasing the CTL content, clearly suggesting that the higher amount of CTL—with respect to HA90—leads to the formation of larger coacervates. Though the hydrodynamic diameter increased, the size distribution curve preserved a unimodal profile in the case of *r*HA 0.25 coacervates, suggesting that the resulting colloids can still be considered as homogeneous from a dimensional point of view (see the PDI values in Table 2). 

## 4. Conclusions

In this contribution, we have reported for the first time an in-depth investigation on the effects of experimental variables on complex coacervation between hyaluronic acid and the lactose-modified chitosan CTL. The present work has demonstrated that parameters such as pH, ionic strength, polymers’ weight ratio and macromolecular dimensions are pivotal factors in modulating the process. The behavior of two oppositely charged polymers was studied when forming binary solutions. A complete miscibility of polysaccharides was achieved at pH 7.4 for all the ionic strengths analyzed due to residual positive charges on CTL. At pH 4.5, an increase of turbidity manifested itself, marking the liquid-liquid phase separation. DLS analyses have shed light on the influence of HAs at different molecular weights on the resulting coacervates. The formulations prepared in the absence of ionic strength displayed a decrement of homogeneity when increasing the molecular weight of HA. Conversely, a low molecular weight HA produced coacervates with good polydispersity, thus acting as true “cross-linkers” for CTL chains. Finally, ζ-potential analyses have shown the possibility of changing coacervates’ surface charge while varying polymers’ weight fractions, with the potential translation of the present system toward the Drug Delivery sector. 

## Figures and Tables

**Table 1:** The intrinsic viscosity and viscosity-average molecular weight of hyaluronic acids used in this study.

HA Sample	[η] (mL/g)	$\bar{M_{v}}$
**HA90**	270	90,000
**HA310**	736	310,000
**HA570**	1210	570,000

**Table 2:** Comparison of size and PolyDispersity Index (PDI) of coacervates prepared with HAs at different molecular weights and weight fractions (*r*HA).

Sample	Size (nm)	PDI
HA90/CTL *r*HA 0.75	255 ± 6	0.28 ± 0.02
HA310/CTL *r*HA 0.75	295 ± 5	0.56 ± 0.05
HA570/CTL *r*HA 0.75	487 ± 83	0.55 ± 0.21
HA90/CTL *r*HA 0.25	978 ± 3	0.27 ± 0.02
